# An Account of an Hemorhagic Disposition Existing in Certain Families

**Published:** 1808-07

**Authors:** John C. Otto

**Affiliations:** Philadelphia


### *An Account of an* Hemorhagic Disposition *existing in certain Families.* By John C. Otto, M. D. *of Philadelphia.*

About seventy or eighty years ago, a woman by the name of Smith, settled in the vicinity of Plymouth, New-Hampshire, and transmitted the following idiosyncrasy to her her descendants. It is one, she observed, to which her family is unfortunately subject, and had been the source not only of great solicitude, but frequently the cause of death. If the least scratch is made on the skin of some of them, as mortal a hæmorrhagy will eventually ensue as if the largest wound is inflicted. The divided parts, in some instances, have had the appearance of uniting, and have shown a kind disposition to heal; and, in others, cicatrization has almost been perfect, when, generally about a week from the injury, an hemorrhagy takes place from the whole surface of the wound, and continues several days, and is then succeeded by effusions of serous fluid; the strength and spirits of the person become rapidly prostrate; the countenance assumes a pale and ghastly appearance; the pulse loses its force, and is increased in frequency; and death, from mere debility, then soon closes the scene. Dr. Rogers attended a lad, who had a slight cut on his foot, whose pulse "was full and frequent" in the commencement of the complaint, and whose blood "seemed to be in a high state of effervescence." So assured are the members of this family of the terrible consequences of the least wound, that they will not suffer themselves to be bled on any consideration, having lost a relation by not being able to stop the discharge occasioned by this operation.

Various remedies have been employed to restrain the hemorrhagies; the bark, astringents used topically and internally, strong styptics, opiates, and, in fact, all those means that experience has found serviceable, have been tried in vain. Physicians of acknowledged merit have been consulted, but have not been able to direct any thing of utility. Those families that are subject to certain complaints are occasionally relieved by medicines that are inefficacious when applied to others; and family receipts are often of greater advantage in restoring them, than all the drugs the materia medica offers for that purpose. A few years since the sulphate of soda was accidentally found to be completely curative of the hemorrhages I have described. An ordinary purging dose, administered two or three days in succession, generally stops them; and, by a more frequent repetition, is certain of producing this effect. The cases in which the most powerful, and apparently the most appropriate remedies have been used in vain, and those in which this mode of treatment has been attended with success, are so numerous, that no doubt can exist of the efficacy of this prescription. The persons who are subject to this hemorrhagic idiosyncrasy, speak of it with the greatest est confidence. Deceptions may take place from accidental coincidence; but when a complaint has often occurred, and been almost uniformly fatal without the administration of a certain medicine, and has constantly yielded when it has been given, scepticism should be silent with regard to its utility. Nor should our inability to account for the fact, upon the theory and principles we have adopted, be conceived a sufficient reason for disbelieving it. An attempt to explain the mode of operation of this valuable remedy might give birth to much speculation. As the affection has been attended with mortality, and there is generally a disposition to give relief as early as possible, experiments have not been made with the other neutral salts to learn their comparative effect; nor have medicines been tried, whose operation might be supposed to be similar. The prescription being known to the whole family, application is rarely made to a physician; and when it is, it is rather with a view of directing him how to proceed, than of permitting him to make a series of trials and observations which might be at the hazard of the life of the patient. The utility of the sulphate of soda cannot arise from its debilitating effects, since it has been found serviceable when the previous depletion has been great, the strength much exhausted, and the system has evidenced symptoms of direct debility. Perhaps time will elucidate its mode of operation, and some general principles may be developed that may be applied to advantage in restraining ordinary hemorrhages; but reasoning upon what has been discovered to be useful in idiosyncracies, and applying it to the general constitution of human nature, must necessarily be vague, and productive of occasional evil. In every case, however, a doubtful remedy is preferable to leaving the patient to his fate. The sulphate of soda has constantly succeeded when administered; but the prescription being in the possession of the Shepard family, the descendants of Smith, and the cases that have been attended by physicians not being very numerous, it is impossible to ascertain the various states of the system in which it has been given, or to form any correct conclusions respecting its manner of acting. No experiments have been made on the blood, to discover if any or what changes take place in it.

It is a surprising circumstance that the males only are subject to this strange affection, and that all of them are not liable to it. Some persons, who are curious, suppose they can distinguish the bleeders (for this is the name given to them) even in infancy; but as yet the characteristic marks are not ascertained sufficiently definite. Although the females are exempt, they are still capable of transmitting it to their male children, as is evidenced by its introduction, and other instances, an account of which I have received from the Hon. Judge Livemore, who was polite enough to communicate to me many particulars upon this subject. This fact is confirmed by Drs. Rogers and Porter, gentlemen of character residing in the neighbourhood, to whom I am indebted for some information upon this curious disposition. When the cases shall become more numerous, it may perhaps be found that the female sex is not entirely exempt; but, as far as my knowledge extends, there has not been an instance of their being attacked.

The persons subject to this hemorrhagic disposition are remarkably healthy, and, when indisposed, they do not differ in their complaints, except in this particular, from their neighbours. No age is exempt, nor does any one appear to be particularly liable to it. The situation of their residence is not favourable to scorbutic affections or disease in general. They live, like the inhabitants of the country, upon solid and nutritious food, and when arrived to manhood, are athletic, of florid complexions, and extremely irascible.

Dr. Rush has informed me, he has been consulted twice in the course of his practice upon this disease. The first time, by a family in York, and the second, by one in Northampton county, in this State. He likewise favoured me with the following account, which he received some years since from Mr. Boardley, of a family in Maryland, afflicted with this idiosyncrasy.

"A. B. of the State of Maryland, has had six children, four of whom have died of a loss of blood from the most trifling scratches or bruises. A small pebble fell on the nail of a fore-finger of the last of them, when at play, being a year or two old; in a short time, the blood issued from the end of that finger, until he bled to death. The physicians could not stop the bleeding. Two of the brothers, still living, are going in the same way; they bleed greatly upon the slightest scratch, and the father looks every day for an accident that will destroy them. Their surviving sister shows not the least disposition to that threatening disorder, although scratched and wounded. The father gave me this account two days since, but I was not inquisitive enough for particulars."

